# TAT-Ngn2 Enhances Cognitive Function Recovery and Regulates Caspase-Dependent and Mitochondrial Apoptotic Pathways After Experimental Stroke

**DOI:** 10.3389/fncel.2018.00475

**Published:** 2018-12-14

**Authors:** Yu Zhao, Jinling Wang, Jiwei Du, Baixiang Li, Xingchun Gou, Jiannan Liu, Lichao Hou, Hanfei Sang, Bin Deng

**Affiliations:** ^1^Department of Anesthesiology, Xiang’an Hospital, Xiamen University, Xiamen, China; ^2^Department of Hygienic Toxicology, Public Health College, Harbin Medical University, Harbin, China; ^3^Department of Anesthesiology, Heilongjiang Provincial Hospital, Harbin, China; ^4^Department of Emergency, Zhongshan Hospital, Xiamen University, Xiamen, China; ^5^Department of Nursing, Xiang’an Hospital, Xiamen University, Xiamen, China; ^6^Shaanxi Key Laboratory of Brain Disorders & Institute of Basic and Translational Medicine, Xi’an Medical University, Xi’an, China; ^7^State Key Laboratory of Cellular Stress Biology, Innovation Center for Cell Signaling Network, School of Life Sciences, Xiamen University, Xiamen, China

**Keywords:** global cerebral ischemia, neurogenin-2, brain-derived neurotrophic factor, transactivator of transcription domain, apoptosis

## Abstract

Neurogenin-2 (Ngn2) is a basic helix-loop-helix (bHLH) transcription factor that contributes to the identification and specification of neuronal fate during neurogenesis. In our previous study, we found that Ngn2 plays an important role in alleviating neuronal apoptosis, which may be viewed as an attractive candidate target for the treatment of cerebral ischemia. However, novel strategies require an understanding of the function and mechanism of Ngn2 in mature hippocampal neurons after global cerebral ischemic injury. Here, we found that the expression of Ngn2 decreased in the hippocampus after global cerebral ischemic injury in mice and in primary hippocampal neurons after oxygen glucose deprivation (OGD) injury. Then, transactivator of transcription (TAT)-Ngn2, which was constructed by fusing a TAT domain to Ngn2, was effectively transported and incorporated into hippocampal neurons after intraperitoneal (i.p.) injection and enhanced cognitive functional recovery in the acute stage after reperfusion. Furthermore, TAT-Ngn2 alleviated hippocampal neuronal damage and apoptosis, and inhibited the cytochrome C (CytC) leak from the mitochondria to the cytoplasm through regulating the expression levels of brain-derived neurotrophic factor (BDNF), phosphorylation tropomyosin-related kinase B (pTrkB), Bcl-2, Bax and cleaved caspase-3 after reperfusion injury *in vivo* and *in vitro*. These findings suggest that the downregulation of Ngn2 expression may have an important role in triggering brain injury after ischemic stroke and that the neuroprotection of TAT-Ngn2 against stroke might involve the modulation of BDNF-TrkB signaling that regulates caspase-dependent and mitochondrial apoptotic pathways, which may be an attractive therapeutic strategy for cerebral ischemic injury.

## Introduction

Global cerebral ischemic injury is commonly observed in clinical conditions such as cardiac arrest and stroke (Tiainen et al., 2015), which mainly attack vulnerable brain regions, such as the CA1 region in the hippocampus (Li et al., 2017), leading to neuronal apoptosis and neurological deficit in memory (Sabri et al., 2013; Zhao et al., 2017). However, there is currently no effective therapy. Therefore, there is still an urgent need to develop novel drug targets and treatment strategies against cerebral ischemic injury.

Studies have reported that neurogenin-2 (Ngn2) is a basic-helix-loop-helix (bHLH) transcription factor that contributes to the identification and specification of neuronal fate during neurogenesis (Andersson et al., 2007; Shaker et al., 2012). Further study showed that transplanted Ngn2-transduced neural precursor cells (NPCs) enhanced cell survival in the adult rat brain and improved hindlimb locomotor function after severe spinal cord injury (Yi et al., 2008). Another study also reported that Ngn2-NPCs transplantation increased cell survival and enhanced structural and functional recovery by attenuating hypoxic-ischemic (HI) brain injury (Lee et al., 2017). In our previous study, we found that Ngn2 plays an important role in alleviating neuronal apoptosis, which can be viewed as an attractive candidate for the treatment of focal cerebral ischemia (Deng et al., 2013). However, the function and mechanism of Ngn2 in mature hippocampal neurons after global cerebral ischemic injury remains unclear.

Neurotrophins, such as brain-derived neurotrophic factor (BDNF), are widely distributed throughout the central nervous system (CNS). The high-affinity receptor of BDNF, tropomyosin-related kinase B (TrkB), can be auto-phosphorylated after BDNF binding to TrkB, which subsequently activates several intracellular signaling pathways exerting neuroprotective effects against cerebral ischemic injury, epilepsy, Alzheimer’s disease and so on (Han et al., 2011; Yang et al., 2018). A previous study reported that Ngn2-transduced human NPCs significantly upregulated the gene expression levels of BDNF, which increased cell survival and enhanced structural and functional recovery in a mouse model of neonatal HI brain injury (Lee et al., 2017). Cheng et al. (2014) reported that transplantation of Ngn2-transduced mesenchymal stem cells (MSCs) decreased apoptotic cells and improved neurological functional recovery by increasing the expression of BDNF against stroke. However, whether Ngn2 plays an important role in BDNF/TrkB expression in neurons after cerebral ischemic injury is still unknown.

To avoid the shortcomings of gene therapy, many reconstructive protein drugs have been introduced for clinical applications (Harada et al., 2012). However, the delivery of therapeutic proteins into the brain has been hampered by the blood-brain barrier (BBB; Lichota et al., 2010). It has been demonstrated that the protein transduction domain (PTD) derived from a human immunodeficiency virus (HIV) transactivator of transcription (TAT) can carry the fusion proteins across cell membranes and the BBB after intraperitoneal (i.p.) injection, which represents a novel and promising strategy for treating experimental brain injury, as reported by us and others (Cook et al., 2012; Deng et al., 2013, 2018).

Therefore, in this study, we attempted to investigate the expression and function of Ngn2 in mature hippocampal neurons in the acute stage after global cerebral ischemic injury. Then, we tested whether BDNF/TrkB signaling pathways were regulated by Ngn2 after ischemic reperfusion injury. After that, the neuroprotection of the recombinant fusion protein TAT-Ngn2 and the possible underlying molecular events were explored in experimental models of stroke. The outcomes of this study provide significant insights into the novel connection between Ngn2 and the expression of BDNF/TrkB signaling pathways implicated in neuronal apoptosis and towards the potential role and mechanism of TAT-Ngn2 against ischemic reperfusion injury.

## Materials and Methods

### Animals

The animals were provided by the Experimental Animal Center of Xiamen University (Xiamen, China). Male C57BL/6 mice weighing 25–30 g were used. During the experiments, all efforts were made to minimize animal suffering and the number of animals used. The animals were housed in a specific pathogen-free environment with free access to sterile laboratory food pellets and water. The experimental protocol was approved by the Ethics Committee for Animal Experimentation of Xiamen University.

### Global Cerebral Ischemia

Bilateral common carotid artery occlusion (BCCAO) was used as a model of global cerebral ischemia (Li et al., 2016). Surgical operations were performed by a person who was blinded to the animal groups. Mice were anesthetized with a mixture of 3% isoflurane and 97% oxygen. A midline incision was made and both the right and left common carotid arteries were carefully separated from the surrounding tissue and the vagus nerve. Cerebral ischemia was induced by clamping both of the arteries with two miniature artery clips. A laser Doppler flowmeter (PeriFlux system 5000, Perimed, Sweden) was used to measure the regional cerebral blood flow (rCBF; 2–3 mm lateral to bregma) from the time of anesthetic induction to 5 min after reperfusion. In our experimental model, only mice whose mean cortical rCBF was reduced to <30% of the preischemic value were included in the data analysis. The clips were removed from both arteries after 20 min of cerebral ischemia (Li et al., 2017). During the surgical procedure, the pericranial temperature was monitored using a temperature probe and maintained at 37.0–37.5°C using a heating pad. After surgery, animals were placed in a warm environment (25°C) to avoid biasing results due to hypothermia.

### The Administration of TAT-Ngn2 *in vivo* Study

TAT-Ngn2 construction, expression and purification were carried out as previously reported (Deng et al., 2013). The protein was dissolved with 0.9% normal saline. Treatments were carried out through i.p. administration of TAT-Ngn2 at 250 mg/kg/injection (only once) in normal adult mice or after reperfusion as previously reported (Deng et al., 2013).

### Behavioral Evaluation

The total motor score (TMS) was used to detect the motor deficits of mice at 48 h after ischemic insult in the BCCAO model. Each mouse was placed on a 10 × 20 cm screen with a 0.2 × 0.2 cm grid. Once the mouse was placed on it, the screen was rotated from 0° (horizontal) to 90° (vertical). The time that the mouse held on to the screen was recorded to a maximum of 15 s. Next, the mouse was placed on the center of a balance beam (1.5 cm in diameter, 50 cm in length), and the time that mouse could stay on it was recorded to a maximum of 15 s. Finally, the mouse was placed on a horizontal rope, and the time the mouse grabbed the rope was recorded to a maximum of 5 s. TMS (the maximal score was 9) was computed from these tests. We performed these tests in a previous study that evaluated global cerebral ischemia in mice (Homi et al., 2003).

Spatial working memory and reference memory were evaluated by the T-maze at 48 h after reperfusion injury. One arm of the maze was designated correct and the other arm was incorrect. The mice were placed at the end of the box and given a 5 s foot shock. The shock continued until the mouse entered the correct arm. The mice were trained until they made one avoidance; that is, they avoided the shock by entering the correct arm. The numbers of trials it took the mice to make their first avoidance response were measured. Details of the methods are described in a previous study (Farr et al., 2014).

The elevated plus-maze test was performed at 48 h after reperfusion injury. The elevated plus-maze consisted of a black plastic apparatus with fours arms (16 cm × 5 cm) set in a cross from a neutral central square (5 cm × 5 cm). The maze was elevated 30 cm above the ground and illuminated from the top. A pretest trial was carried out on the first day. The mouse was placed on the open arm to see if it moved to the enclosed arm. If the mouse failed to enter the enclosed arm, it was placed on the enclosed arm for an additional 60 s, and the transfer latency (TL) 1 value was recorded as 90 s. If the mouse entered the enclosed arm within 90 s, the time the mouse took was recorded as TL1. The retention trial was performed 24 h later in the same way as the first trial, and the TL was recorded as TL2. If the mouse did not enter the enclosed arm within 90 s, the test was stopped and the TL2 was recorded as 90 s. We recorded the TL2 of each mouse after the test (Li et al., 2016).

The latency and number of errors were recorded during a step-down passive avoidance test. The latency to step-down was recorded at 24 h after reperfusion injury. Before the start of the training trial, mice were habituated to the chamber for 3 min. Mice jumped to a platform when electric shocks were given; the shocks lasted 5 min. The retention of memory was tested after 24 h in a test trial; mice were placed on the same platform used in the training trial. The step-down latency and number of errors within 5 min were recorded. If the mice did not jump down from the platform within 5 min, the latency was recorded as 300 s. Additional details of the test situation are described in a previous study (Zhang et al., 2014; Li et al., 2016).

### Immunohistochemistry Staining

To explore the delivery and distribution of TAT-Ngn2 after i.p. injection, anti-6× His immunofluorescence staining was assessed at 6 h and 48 h after i.p. injection of TAT-Ngn2 (250 mg/kg). At 6 and 48 h after i.p. injection of TAT-Ngn2, the mice were anesthetized with sodium pentobarbital (40 mg/kg, i.p.) and perfused transcardially with 10 mL saline and then 50 mL of 4% paraformaldehyde (PFA) in 0.1 mol/L phosphate buffer (pH7.4). The brains were dissected immediately and placed in 0.1 mol/L phosphate buffer containing 20% sucrose overnight at 4°C. After that, the brain samples containing hippocampus were serially cut into coronal 16-μm thick sections on frozen microtome. The sections were washed with PBS, permeabilized in 0.3% (v/v) Triton X-100 and 1% (w/v) bovine serum albumin (BSA) in PBS for 30 min, blocked for 30 min in blocking buffer (4% (v/v) normal mouse serum and 1% (w/v) BSA), and incubated for 16 h in blocking buffer containing mouse monoclonal antibody against (His)_6_ (1:1,000; Abcam, USA) and rabbit monoclonal antibody against NeuN (1:20,000; Abcam, USA). Sections were further washed with PBS and incubated for 1 h in blocking buffer containing secondary antibody (FITC-labeled goat anti-mouse IgG; Molecular Probes, USA, and Alexa Fluor 594-conjugated anti-rabbit IgG; Molecular Probes, USA). Images were captured using an Olympus FluoView FV10i confocal laser scanning microscope (Olympus Corp., Shinjuku, Tokyo, Japan; Chen et al., 2008).

### Nissl, Fluoro-Jade C and TUNEL Staining

At 48 h after reperfusion, the mice were deeply anesthetized by i.p. injection of sodium pentobarbital (40 mg/kg) and perfused using 0.1 M PBS followed by 4% PFA in PBS. The brain tissues were removed, placed in 20% (w/v) sucrose and sectioned in 16 μm slices, 3.5 mm posterior to bregma. The sections were processed for Nissl, Fluoro-Jade C (FJC) and terminal deoxynucleotidyl transferase-mediated dUTP-biotin nick end labeling (TUNEL) staining. Nissl staining (Beyotime Institute of Biotechnology, China) was performed to detect Nissl bodies in the cytoplasm of surviving neurons. The integrated optical density/area of the staining in each group was acquired by two blinded investigators using ImagePro Plus 5.1 software (Media Cybernetics, Inc., Bethesda, MD, USA). Details of the methods are described in a previous study (Wang et al., 2011).

FJC staining was performed as previously described (Bian et al., 2007; Wang L. et al., 2008). The protocol was as follows: (1) pretreatment with alcohol-sodium hydroxide mixture, the sections were immersed in a solution containing 1% sodium hydroxide in 80% alcohol for 5 min, followed by 70% alcohol and distilled water each for 2 min; (2) then, the sections were transferred into a solution of 0.06% potassium permanganate for 10 min and rinsed in distilled water for 2 min; (3) after that, the sections were immersed into a 0.0001% solution of FJC dye (Chemicon, Temecula, CA, USA) dissolved in 0.1% acetic acid vehicle (pH 4) and stained for 20 min; (4) the slides were washed three times in distilled water each for 1 min and left to dry overnight in darkness at room temperature; and (5) sections were air-dried, dehydrated in ethanol, cleared in xylene and coverslipped with DPX. Finally, the FJC-stained sections were examined under an epifluorescence microscope or a laser scanning confocal microscope (Olympus, BX-60). The FJC-positive stain exhibited a strong green color using a filter system that was the same as that used for activating fluorescein.

For detection of *in situ* DNA fragmentation, TUNEL staining was performed *in vivo* and *in vitro* using an *in situ* Cell Death Detection Kit (Roche Diagnostics, Mannheim, Germany) as described previously (Yu et al., 2012). Evidence suggested that C57BL/6 mice are particularly susceptible to neuronal damage in the CA1 region of the hippocampus after transient global ischemia. The pyramidal neurons in the CA1 are more susceptible to ischemic reperfusion injury. The total number of TUNEL positive neurons in the CA1 region was counted in three different fields for each section by two blinded investigators using Image-Pro plus 5.1 software (Media Cybernetics, Inc., Bethesda, MD, USA).

### Electron Microscopy Analysis

The ultrastructure of the neurons in the hippocampus CA1 area was observed at 48 h after reperfusion. As described previously (Deng et al., 2014), the mice were anesthetized i.p. with 1% sodium pentobarbital (50 mg/kg body weight) and perfused transcardially with 150 ml of warm saline followed by 500 ml of ice-cold fixative for 30 min. A mixture of 1% PFA and 2% glutaraldehyde in 0.1 M sodium phosphate buffer (pH 7.4) was used as a fixative. A segment of the hippocampus was then removed and postfixed by immersion in the same fixative for 2 h at 4°C. We then prepared ultrathin sections following standard procedures and observed the sections using a JEM-100SX electron microscope.

### Primary Hippocampal Neuron Culture

Primary hippocampal neurons were cultured as described previously (Deng et al., 2013). The purity of neurons was determined by immunocytochemistry for βIII-tubulin, which indicated that 95% of cells in the cultures were βIII-tubulin (1:250; Millipore, Temecula, CA, USA) positive (data not shown). Then, the transduction ability of TAT-Ngn2 was assayed by an immunofluorescence analysis (Wang et al., 2009). In brief, the neurons were cultured with TAT-Ngn2 (125 μg/l) for 24 h, washed three times with PBS after fixation in 4% PFA (Dietz et al., 2002). Then incubated simultaneously with primary antibodies: anti-6× His antibody (1:2,000; Abcam, Cambridge, MA, USA) and βIII-tubulin (1:250; Millipore, Temecula, CA, USA) overnight at 4°C. The neurons were washed three times with PBS and then incubated with two secondary antibodies: FITC-labeled goat anti-rabbit IgG (1:200; Vector, Burlingame, CA, USA) and Alexa 594-labeled donkey anti-mouse IgG (1:500; Molecular Probes, Rockford, IL, USA) for 1 h at 37°C. Nuclei were visualized by DAPI staining. Images were viewed with a fluorescence microscope (BX60; Olympus). These measurements were made on cells present in six randomly selected fields in each experiment and repeated in at least three independent cultures.

### Oxygen Glucose Deprivation (OGD) Model

To test the neuroprotective function of TAT-Ngn2 against ischemia injury *in vitro*, the oxygen glucose deprivation (OGD) model was used as described (Zeng et al., 2012). In brief, we designed several groups: normal group, OGD group, OGD+control virus group, OGD+Ngn2 virus group, and OGD+TAT-Ngn2 (125 μg/L) group. The hippocampal neurons were cultured first in a neurobasal medium without glucose (Invitrogen, Carlsbad, CA, USA) in a humidified incubator filled with an anoxic gas mixture (5% CO_2_ and 95% N_2_) at 37°C for 1 h and then in normal medium in the presence or absence of control virus, *ngn2* virus or TAT-Ngn2.

### Preparation of TAT-Ngn2 Fusion Protein and Virus-Mediated Overexpression of *ngn2* Gene

TAT-Ngn2 fusion protein construction, expression and purification were produced as previously reported (Deng et al., 2013). Briefly, the mouse Ngn2 cDNA sequence was synthesized by Genscript Co. (Nanjing, China) and included NcoI and XhoI site sequences at 5’ and 3’. The synthesized product was cloned into the NcoI/XhoI sites of the pTAT-HA vector. The final expression plasmid was named pTAT-Ngn2 and contained a (His)6 tag, a TAT sequence (YGRKKRRQRRR) and a Ngn2 sequence. The pTAT-Ngn2 was transformed into the *E. coli* strain BL21 (DE3). Protein production was induced with 100 mM isopropyl β-D-1-thiogalactopyranoside (TaKaRa, Tokyo, Japan) and purified by Ni-NTA agarose chromatography (Merck, Darmstadt, Germany). The size and purity were confirmed by sodium dodecyl sulfate-polyacrylamide gel electrophoresis (SDS-PAGE). Lentivirus transfection of neurons was performed as previously reported (Fierro et al., 2011; Cho et al., 2012). The vector lenti-Ngn2 contains a neomycin resistance gene for establishing a stable cell line and a coral GFP gene for tracking transfection efficiency, driven by the CMV promoter and SV40 promoter, respectively. To infect the cells, 1 × 10^5^ neurons were exposed to 10 × 10^7^ virus particles for 48 h.

### MTT Assay and Measurement of LDH Release *in vitro*

At 48 h after OGD injury, the cell viability was assessed by the methyl thiazolyl tetrazolium (MTT) assay as described (Zeng et al., 2012). Briefly, the culture medium was changed to DMEM containing 0.5 g/L MTT. After incubation for 4 h at 37°C, the supernatant was discarded, and the cells were mixed thoroughly with dimethylsulfoxide (DMSO, 100 μL/well). When the crystals were dissolved, the optical density absorbance values in each well were measured with an Elx800 plate reader (BioTek Instruments Inc., Winooski, VT, USA) at 570 nm. Cell viability is directly proportional to the absorbance value.

We also assessed lactate dehydrogenase (LDH) release, an indicator of neuronal injury, with a commercially available kit (Cheng et al., 2009). The leakage of LDH was measured kinetically by following the changes in NADH (0.01 mM) at 340 nm using 0.7 mM sodium pyruvate in 0.05 M Tris–HCl buffer (pH 7.4, 25°C). LDH release was calculated as the percentage of LDH in the medium vs. total LDH in the cells.

### Western Blot Analysis

We analyzed the expression of Ngn2 NGF, BDNF, phosphorylation TrkB (p-TrkB), total TrkB, cytochrome C (CytC) in the cytoplasm (C CytC), CytC in the mitochondria (M CytC), active caspase 3, Bcl-2 and Bax by western blotting *in vivo* and *in vitro* as described previously. The total protein concentration of the tissues or cells was analyzed with a BCA kit (Sigma, CA, USA). The blots were probed with mouse monoclonal anti-Ngn2 rabbit antibody (1:200; Abcam, USA), mouse monoclonal antibody anti-6-His (1:1,000; Abcam, USA), monoclonal anti-NGF (1:2,000, Abcam, USA), mouse monoclonal anti-BDNF (1:2,000, Abcam, USA), rabbit monoclonal anti-p-TrkB or TrkB (1:1,000, Affinity, USA), rabbit polyclonal anti-CytC (1:1,000, Abcam, USA), rabbit antibody against cleaved (active) caspase-3 (1:1,000; Cell Signaling Technology, Beverly, MA, USA), mouse monoclonal antibodies against Bcl-2 or Bax (1:1,000, Santa Cruz, CA, USA), Cytochrome C oxidase IV (COX IV) and β-actin (1:2,000, Anbo, USA). Subsequently, the blots were probed with a horseradish peroxidase (HRP)-conjugated goat secondary antibody against rabbit or mouse IgG (1:1,000, Abcam, USA). Detection and quantitation were performed with a Typhoon 9400 Variable Mode Imager (GE Healthcare) and Lumi-Light Western Blotting Substrate (Roche Diagnostics) for HRP-labeled blots.

### Real-Time PCR

The levels of *ngn2* mRNA in the cerebral cortex, hippocampus and corpus striatum was assessed at 12 h, 24 h and 48 h after reperfusion in the global cerebral ischemic injury model. PCR was performed under the following thermal cycling conditions: one cycle at 95°C for 5 min; 25 cycles at 95°C for 30 s, 60°C for 60 s, and 72°C for 30 s and one cycle at 72°C for 10 min. Melt-curve analysis was used to identify different reaction products, including nonspecific products. The following primers for RT-PCR were designed by the TaKaRa corporation: Ngn2: F: 5′-CATTTGCAATGGCTGGCAT C-3′, R: 5′-CAATAGGCATTGTGACGAATCTGG-3′; GAPDH::5′-TGTGTCCGTC GTGGATCTGA-3′, R: 5′-TTGCTGTTGAAGTCGCAGGAG-3′. Each sample was tested in triplicate. Samples were obtained from three independent experiments and analyzed for relative gene expression data using the 2^−ΔΔCt^ method.

### Statistical Analysis

All data were collected and analyzed by researchers blinded to the surgery and reagents that were used. SPSS 18.0 for Windows software (SPSS, Inc., Chicago, IL, USA) was used to conduct the statistical analyses. All values are presented as the mean ± standard error of the mean (SEM). The statistics in this study were based on Student’s *t*-test and one-way or two-way analysis of variance (ANOVA) with Bonferroni’s correction. Values of *P* < 0.05 were considered statistically significant. Statistical analyses were performed with GraphPad Prism software (GraphPad Software, La Jolla, CA, USA).

## Results

### Expression of Ngn2 in the Cerebral Ischemic Reperfusion Injury Model: *in vivo* and *in vitro* Studies

**Figure 1:**
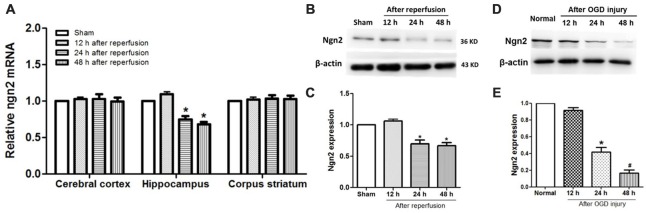
Expression of neurogenin-2 (*ngn2*) mRNA and Ngn2 protein in both *in vivo* and *in vitro* studies. **(A)** Expression of Ngn2 at the mRNA level in the cerebral cortex, hippocampus and corpus striatum was quantified using quantitative real-time PCR (qPCR) at 12 h, 24 h and 48 h after reperfusion in the global cerebral ischemic injury model (*n* = 8 for each group, *P < 0.05 vs. the sham group, the data were analyzed using one-way ANOVA with Dunnett’s test). **(B,C)** Expression of Ngn2 at the protein level in the hippocampus was quantified using western blotting at 12 h, 24 h and 48 h after reperfusion in the global cerebral ischemic injury model (*n* = 8 for each group, *P < 0.05 vs. the sham group, the data were analyzed using one-way ANOVA with Dunnett’s test). **(D,E)** Expression of Ngn2 in hippocampal neurons was detected using western blotting at 12 h, 24 h and 48 h after oxygen glucose deprivation (OGD) injury. Band densities were measured using the ImageJ program and normalized to β-actin (*n* = 6 for each group, *P < 0.05 vs. the sham group, ^#^*P* < 0.05 vs. 12 h after reperfusion group, the data were analyzed using one-way ANOVA with Dunnett’s test).

The temporal and spatial expression pattern of endogenous Ngn2 in the cerebral cortex, hippocampus and corpus striatum was assessed at 12 h, 24 h and 48 h after reperfusion in the global cerebral ischemic injury model. As shown in Figures 1A–C, compared with the sham group, the expression of Ngn2 decreased at 24 h and 48 h after reperfusion in the hippocampus at both the mRNA and protein levels (*P* < 0.05). Then, the expression of Ngn2 in the hippocampal neurons was detected at 12 h, 24 h and 48 h after OGD injury, as shown in Figures 1D,E. The results showed that the expression of Ngn2 decreased 24 h after OGD compared with the normal group (*P* < 0.05). Moreover, the expression of Ngn2 decreased 48 h after OGD compared with 24 h after OGD (*P* < 0.05). The downregulation of Ngn2 expression in the hippocampus in the acute stage after ischemic injury indicated that Ngn2 represents a potential target for stroke therapy.

### TAT-Ngn2 Enhanced Cognitive Functional Recovery

**Figure 2:**
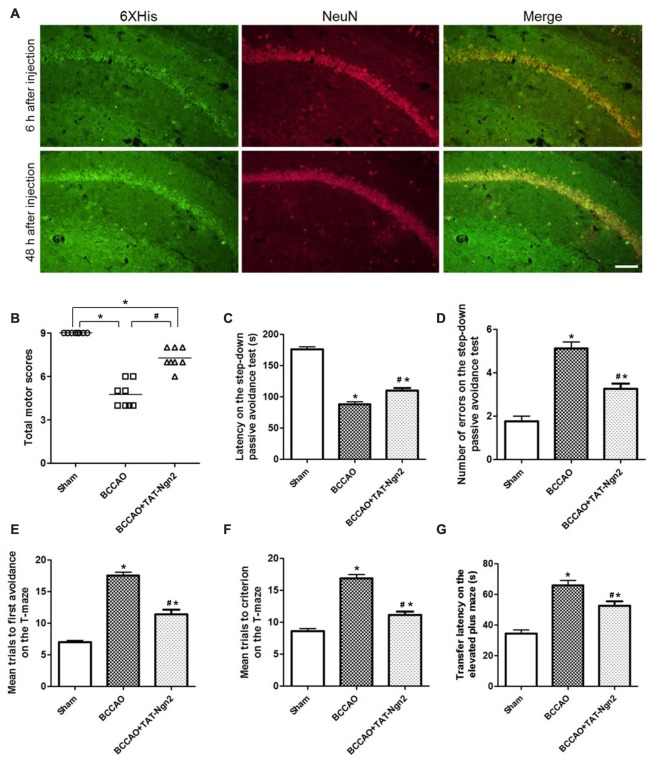
Intraperitoneal (i.p.) injection of transactivator of transcription (TAT)-Ngn2 improved cognitive functional recovery. **(A)** The delivery and distribution of TAT-Ngn2 fusion proteins in the hippocampus. Double immunofluorescence histochemical staining was performed at 6 h and 48 h after i.p. administration of TAT-Ngn2 (250 mg/kg; *n* = 6 for each group). Scale bar = 50 μm. **(B)** Total motor scores (TMSs) were recorded in each animal at 48 h after reperfusion (*n* = 8 for each group, nine serial sections were acquired per mouse in the same area, serial five sections in the middle of nine serial sections were observed and analyzed per animal). **(C)** The latency in the step-down passive avoidance test was measured in each animal at 24 h after reperfusion (*n* = 8 for each group). **(D)** The number of errors in the step-down passive avoidance test was measured in each animal at 48 h after reperfusion (*n* = 8 for each group). **(E)** Mean trials to the first avoidance response on the T-maze were measured in each animal at 48 h after reperfusion (*n* = 8 for each group). **(F)** Mean trials to criterion on the T-maze were measured in each animal at 48 h after reperfusion (*n* = 8 for each group). **(G)** The transfer latency (TL) on the elevated plus maze was measured in each animal at 48 h after reperfusion (*n* = 8 for each group; *P < 0.05 vs. the sham group, ^#^*P* < 0.05 vs. the bilateral common carotid artery occlusion (BCCAO) group). The data from the behavioral experiments were analyzed by one-way analysis of variance followed by Tukey’s multiple comparison tests.

To explore the delivery and distribution of TAT-Ngn2 after i.p. injection, anti-6× His immunofluorescence staining was assessed at different time points after i.p. injection. As shown in Figure 2A, the NeuN proteins colocalized with anti-6× His proteins in the hippocampus. Moreover, the anti-6× His immunofluorescence staining in the hippocampus at 48 h after i.p. injection was more abundant than that at 6 h after i.p. injection. The results revealed that TAT-Ngn2 can be effectively transported and incorporated into the hippocampal neurons after i.p. injection.

At 48 h after reperfusion, the TMS was measured to detect motor deficits. As shown in Figure 2B, the TMS in the BCCAO group was lower than that in the sham group at 48 h after reperfusion (*P* < 0.05). In contrast, the TMS in the BCCAO+TAT-Ngn2 group was significantly higher than that in the BCCAO group (*P* < 0.05). To measure the learning and memory retention ability of mice, the latency and number of errors in the step-down passive avoidance test were examined. As shown in Figures 2C,D, the results showed that the latency to step-down in the BCCAO+TAT-Ngn2 group was increased compared with the BCCAO group (*P* < 0.05) at 24 h after reperfusion. Meanwhile, the number of errors in the step-down task in the BCCAO+TAT-Ngn2 group was decreased compared with the errors in the BCCAO group (*P* < 0.05) at 48 h after reperfusion. Then, the spatial working memory and reference memory were tested in the T-maze at 48 h after reperfusion. As shown in Figures 2E,F, the mice in the BCCAO+TAT-Ngn2 group took fewer trials to make first avoidance response during acquisition and fewer trials to reach the criterion for retention compared with the BCCAO group (*P* < 0.05). The elevated plus maze test was used to test the acquisition and consolidation of memory. The results showed that the TL on the elevated plus maze was shorter in the BCCAO+TAT-Ngn2 group compared with the BCCAO group (*P* < 0.05; Figure 2G) at 48 h after reperfusion. Taken together, these results indicated that TAT-Ngn2 treatment was capable of enhancing the recovery of cognitive function in the acute stage after reperfusion.

### TAT-Ngn2 Alleviated Hippocampal Neuronal Damage and Apoptosis *in vivo*

**Figure 3:**
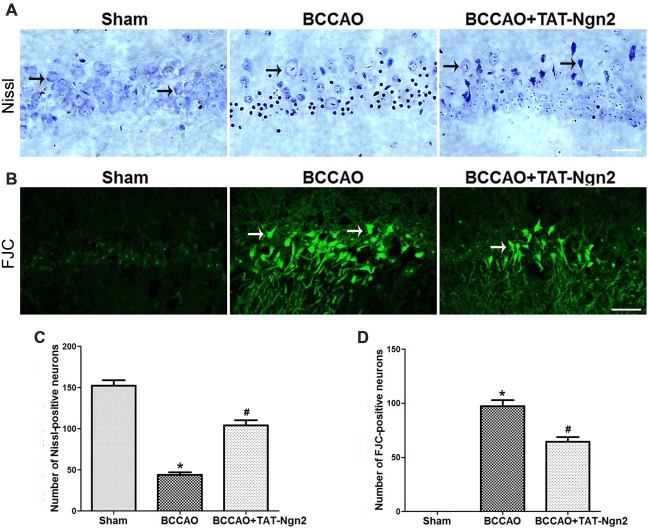
Effect of TAT-Ngn2 on neuronal damage and degeneration in the CA1 region of the hippocampus of mice after BCCAO. **(A)** Representative Nissl staining was performed in the CA1 region of the hippocampus 48 h after reperfusion (*n* = 6 for each group, nine serial sections were acquired per mouse in the same area, serial five sections in the middle of nine serial sections were observed and analyzed per animal). The black arrows indicate the Nissl-positive neurons. Scale bar = 50 μm. **(B)** Representative neuronal degeneration in the CA1 region of the hippocampus was shown by fluoro-jade C (FJC) staining (green) 48 h after reperfusion (*n* = 6 for each group; nine serial sections were acquired per mouse in the same area, serial five sections in the middle of nine serial sections were observed and analyzed per animal). The white arrows indicate the FJC-positive neurons. Scale bar = 50 μm. **(C)** Statistical analysis of the number of Nissl-positive neurons in the CA1 region (*P < 0.05 vs. sham group, *P < 0.05 vs. BCCAO). **(D)** Statistical analysis of the number of FJC-positive neurons in the CA1 region (*P < 0.05 vs. sham group, ^#^*P* < 0.05 vs. BCCAO). The data were analyzed using Student’s *t*-test.

**Figure 4:**
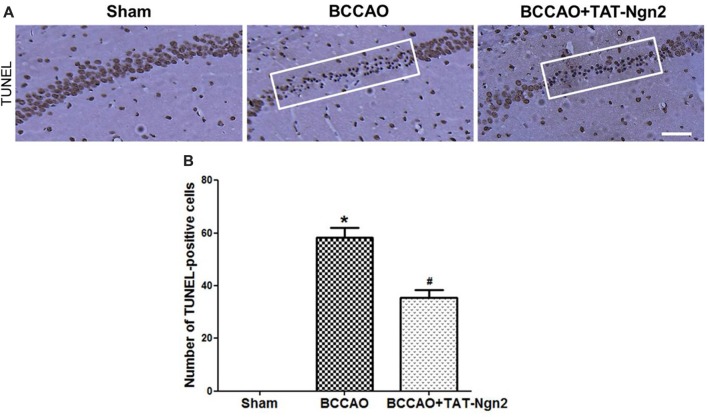
Effect of TAT-Ngn2 on neuronal apoptosis in the CA1 region of the hippocampus of mice after BCCAO. **(A)** Neuronal apoptosis in the CA1 region of the hippocampus is shown by terminal deoxynucleotidyl transferase-mediated dUTP-biotin nick end labeling (TUNEL) staining at 48 h after reperfusion. The rectangular highlighted box was the observation area (*n* = 6 for each group, nine serial sections were acquired per mouse in the same area, serial five sections in the middle of nine serial sections were observed and analyzed per animal). Scale bar = 50 μm. **(B)** Statistical analysis of the number of TUNEL-positive cells in the CA1 region (*P < 0.05 vs. sham group, ^#^*P* < 0.05 vs. BCCAO). The data were analyzed using Student’s *t*-test.

Nissl staining was used to determine the effect of TAT-Ngn2 on hippocampal neuronal damage 48 h after reperfusion. The results showed that the number of Nissl-positive neurons increased in the hippocampus CA1 region in the BCCAO+ TAT-Ngn2 group compared to the BCCAO group (*P* < 0.05; Figures 3A,C). FJC staining was used to determine the effect of TAT-Ngn2 on hippocampal neuronal degeneration at 48 h after reperfusion. The number of FJC-positive neurons was lower in the BCCAO+TAT-Ngn2 group than in the BCCAO group (*P* < 0.05; Figures 3B,D). Then, TUNEL staining was used to identify apoptotic cells. The results showed that the number of TUNEL-positive cells was lower in the BCCAO+TAT-Ngn2 group than in the BCCAO group (*P* < 0.05; Figures 4A,B). These results suggested that TAT-Ngn2 enhanced cognitive functional recovery and attenuated hippocampal neuronal damage and apoptosis.

### TAT-Ngn2 Regulated the Expression of BDNF, the Level of Phosphorylation of TrkB and Mitochondrial CytC Leakage in the Hippocampus *in vivo*

**Figure 5:**
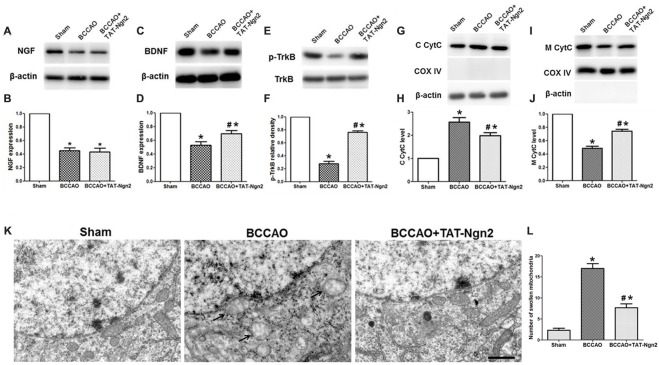
Effect of TAT-Ngn2 on the expression of NGF, brain-derived neurotrophic factor (BDNF), the level of phosphorylation of tropomyosin-related kinase B (TrkB) and mitochondrial cytochrome C (CytC) leakage *in vivo* (*n* = 6 for each group). **(A,C)** Western blotting showing NGF and BDNF expression in the hippocampus at 48 h after reperfusion. **(B,D)** Statistical analysis of NGF and BDNF expression in every group. **(E)** Western blotting showing the p-TrkB expression in the hippocampus at 48 h after reperfusion. **(F)** Statistical analysis of p-TrkB expression in every group. **(G,I)** Western blotting showing the level of CytC in the cytoplasm (C CytC) and CytC in the mitochondria (M CytC) in the hippocampus at 48 h after reperfusion. **(H,J)** Statistical analysis of C CytC and M CytC levels in every group. Band densities were measured using the ImageJ program and normalized to COX IV or to β-actin (*P < 0.05 vs. sham group, ^#^*P* < 0.05 vs. BCCAO group. The data from the Western blotting studies were analyzed using one-way ANOVA with Dunnett’s test). **(K)** Electron microscopic image of the ultrastructure of hippocampal neurons at 48 h after reperfusion. Arrowheads indicate swollen mitochondria in the cytoplasm. The mitochondria swelled to a spherical shape. Scale bar = 1 μm. **(L)** Statistical analysis of the number of swollen mitochondria in every group (*n* = 6 for each group, serially five sections were observed and analyzed per animal; *P < 0.05 vs. sham group, ^#^*P* < 0.05 vs. BCCAO group). The data were analyzed by one-way analysis of variance followed by Tukey’s multiple comparisons tests.

As shown in Figures 5A–F, western blotting revealed that the expression of NGF and BDNF and the level of phosphorylated TrkB decreased in the hippocampus in the BCCAO group compared with the sham group at 48 h after reperfusion (*P* < 0.05). The expression of BDNF and the level of phosphorylation of TrkB increased in the hippocampus in the BCCAO+TAT-Ngn2 group compared with the BCCAO group (*P* < 0.05). However, TAT-Ngn2 had no effect on the expression of NGF. Moreover, as shown in Figures 5G–J, the level of CytC in the cytoplasm in the hippocampus in the BCCAO+TAT-Ngn2 group was less than that in the BCCAO group (*P* < 0.05). In contrast, the level of CytC in the mitochondria in the hippocampus in the BCCAO+TAT-Ngn2 group was higher than that in the BCCAO group (*P* < 0.05).

At 48 h after reperfusion, the ultrastructure of the neurons in the hippocampus CA1 area was disrupted; it appeared that the nuclear membrane had folded, the chromatin moved to the edge, near the nuclear membrane, and then was being degraded. Additionally, the mitochondria were obviously swelling in the cytoplasm, and many mitochondria were spherical. Some mitochondria cristae fractured. The number of swollen mitochondria was higher in the reperfusion group than in the sham group. In contrast, the number of swollen mitochondria was lower in the BCCAO+TAT-Ngn2 group than in the BCCAO group (*P* < 0.05; Figures 5K,L). The results indicated that the damage to structures at the ultrastructural level, especially in the mitochondria, was significantly alleviated with TAT-Ngn2 treatment.

### TAT-Ngn2 or *ngn2* Gene Overexpression Enhanced Neuron Survival and Decreased Cell Apoptosis After OGD Injury *in vitro*

**Figure 6:**
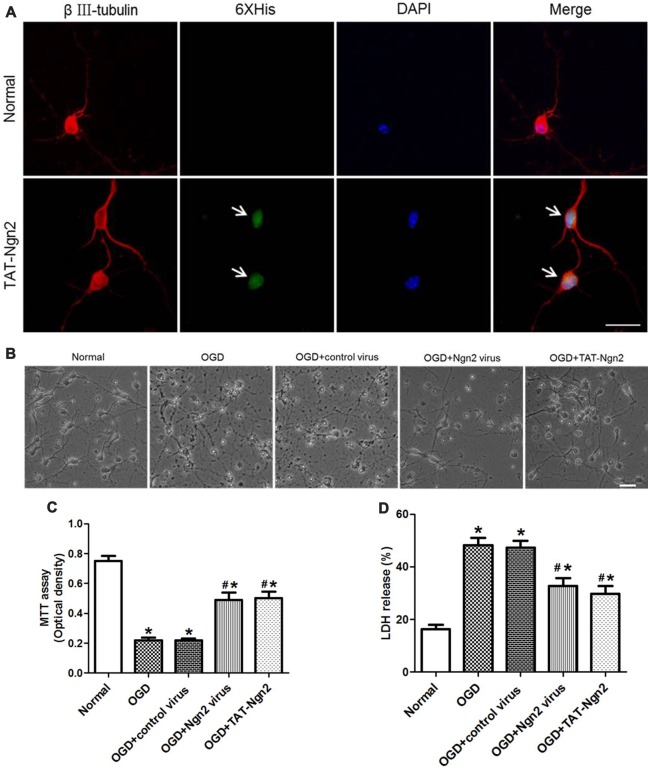
Effect of TAT-Ngn2 or *ngn2* overexpression on cell viability and lactate dehydrogenase (LDH) release after exposure to OGD injury (*n* = 6 for each group). **(A)** Transduction effect of TAT-Ngn2 into hippocampal neurons. Triple immunofluorescence staining was performed 24 h after TAT-Ngn2 incubation with hippocampal neurons. The neurons were detected by double-labeling with βIII-tubulin-positive as red and 6× His-positive as green. The nucleus was counterstained with DAPI (blue). Scale bars = 50 μm. **(B)** The cultured primary hippocampal neurons in all groups under light microscope at 48 h after exposure to OGD. Scale bars = 50 μm. **(C)** Cell viability was detected by an MTT assay at 48 h after exposure to OGD. The values are presented as the mean ± SEM of each group and were analyzed by one-way analysis of variance followed by Tukey’s multiple comparisons test (*P < 0.05 vs. normal group, ^#^*P* < 0.05 vs. OGD group). **(D)** LDH release was detected at 48 h after exposure to OGD. The normal group was defined as 100%. The values are presented as the mean (% of normal) ± SEM of each group and were analyzed by one-way analysis of variance followed by Tukey’s multiple comparisons tests (*P < 0.05 vs. normal group, ^#^*P* < 0.05 vs. OGD group).

To assess the effect of TAT-Ngn2 *in vitro*, TAT-Ngn2 was incubated with the hippocampal neurons for 24 h. The triple immunofluorescence staining result showed that TAT-Ngn2 transduced the cells, whereas fluorescence signals were absent in cells in the normal group (Figure 6A). These results indicated that the TAT-mediated Ngn2 fusion protein had the ability to transduce hippocampal neurons.

The MTT assay revealed that the cell viability in the OGD+Ngn2 virus group and OGD+TAT-Ngn2 group was higher than that in the OGD group or in the OGD+control virus group (*P* < 0.05; Figures 6B,C). Moreover, the cell viability in the OGD+TAT-Ngn2 group was higher than that in the OGD+Ngn2 virus group at 48 h after re-oxygenation/sugar (*P* < 0.05; Figures 6B,C). Then, an LDH release assay was performed to confirm the protective effect of TAT-Ngn2. The results showed that the LDH release in the OGD+Ngn2 virus group and the OGD+TAT-Ngn2 group was lower than that in the OGD group or in the OGD+control virus group (*P* < 0.05; Figure 6D). The results also showed that TAT-Ngn2 attenuated LDH release more effectively than *ngn2* gene overexpression (*P* < 0.05; Figure 6D).

**Figure 7:**
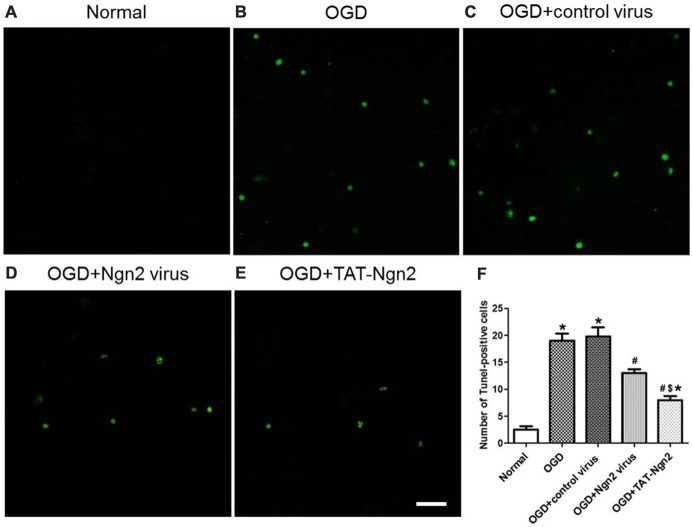
Effect of TAT-Ngn2 or *ngn2* gene overexpression on neuronal apoptosis in neurons exposed to OGD injury (*n* = 6 for each group). **(A–E)** Representative tunel staining of hippocampal neurons 48 h after exposure to OGD injury. The TUNEL-positive neurons are represented in green. Scale bar = 100 μm. **(F)** Statistical analysis of the number of TUNEL-positive cells in each group (*P < 0.05 vs. normal group, ^#^*P* < 0.05 vs. OGD group, ^$^*P* < 0.05 vs. OGD+Ngn2 virus group). The data were analyzed by one-way analysis of variance followed by Tukey’s multiple comparisons test.

**Figure 8:**
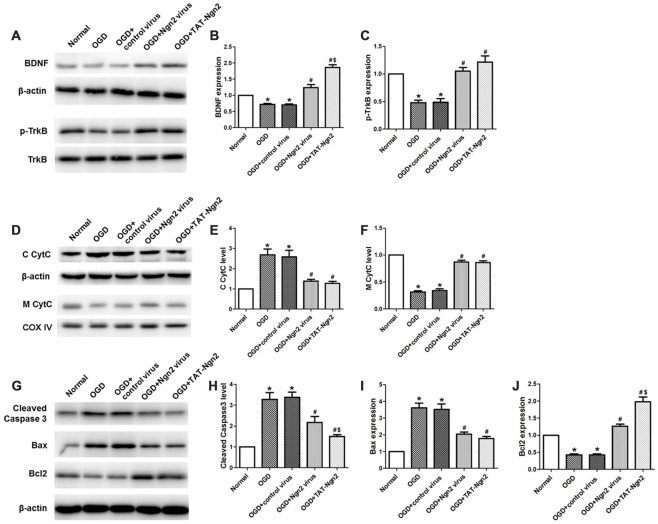
Effect of TAT-Ngn2 on the expression of BDNF, the level of phosphorylation of TrkB and mitochondrial CytC leakage *in vitro* (*n* = 6 for each group). **(A)** Western blotting showing BDNF, p-TrkB expression in cultured primary hippocampal neurons 48 h after reperfusion. **(B,C)** Statistical analysis of BDNF, p-TrkB expression in every group. **(D)** Western blotting showing the level of C CytC and M CytC in the cultured primary hippocampal neurons at 48 h after reperfusion. **(E,F)** Statistical analysis of C CytC and M CytC levels in every group. Band densities were measured using the ImageJ program and normalized to COX IV or to β-actin (*P < 0.05 vs. normal group, ^#^*P* < 0.05 vs. OGD group, ^$^*P* < 0.05 vs. OGD+Ngn2 virus group). **(G)** Representative western blotting of active caspase-3, Bax and Bcl-2 in cultured primary hippocampal neurons 48 h after exposure to OGD injury. **(H–J)** Quantification of relative changes in active caspase-3, Bax and Bcl-2 expression (*P < 0.05 vs. normal group, ^#^*P* < 0.05 vs. OGD group, ^$^*P* < 0.05 vs. OGD+Ngn2 virus group). The data were analyzed using one-way ANOVA with Dunnett’s test.

To investigate the role of Ngn2 in neuronal apoptosis after OGD injury, TUNEL staining was used to assess apoptosis at 48 h after exposure to OGD. The number of TUNEL-positive cells in the OGD group was higher than that in the normal group (*P* < 0.05). In contrast, the number of TUNEL-positive cells in the OGD+Ngn2 virus group and OGD+TAT-Ngn2 group was lower than that in the OGD group or in the OGD+control virus group (*P* < 0.05; Figures 7A–F). As shown in Figures 8G–J the level of cleaved (active) caspase-3 and the expression of Bax increased and the expression of Bcl-2 decreased in the OGD group compared with the normal group (*P* < 0.05). In contrast, the level of cleaved (active) caspase-3 and the expression of Bax decreased, while the expression of Bcl-2 increased, in the OGD+Ngn2 virus group and OGD+TAT-Ngn2 group compared with that in the OGD group or in the OGD+control virus group (*P* < 0.05). These results suggested that TAT-Ngn2 or *ngn2* gene overexpression attenuated neuronal apoptosis after OGD injury and regulated the apoptosis-related proteins cleaved caspase-3, Bax and Bcl-2.

### TAT-Ngn2 or *ngn2* Gene Overexpression Regulated the Expression of BDNF, the Level of Phosphorylation of TrkB and Mitochondrial CytC Leakage *in vivo*

As shown in Figures 8A–F, western blotting results revealed that the expression of BDNF and the level of TrkB phosphorylation decreased in the OGD group compared with the normal group at 48 h after OGD (*P* < 0.05). The expression of BDNF and the level of phosphorylation of TrkB increased in the OGD+Ngn2 virus group and the OGD+TAT-Ngn2 group compared with the levels in the OGD group or in the OGD+control virus group (*P* < 0.05). The levels of CytC in the cytoplasm in the OGD+Ngn2 virus group and OGD+TAT-Ngn2 group was less than the levels in the OGD group or in the OGD+control virus group (*P* < 0.05). In contrast, the level of CytC in the mitochondria in the OGD+Ngn2 virus group and the OGD+TAT-Ngn2 group was higher than these levels in the OGD group or in the OGD+control virus group (*P* < 0.05). These findings suggested that TAT-Ngn2 alleviated neuronal apoptosis and inhibited the CytC leak from the mitochondria to the cytoplasm, which may be one of the mechanisms through which TAT-Ngn2 enhanced cognitive function recovery in the acute stage after stroke.

## Discussion

In the present study, we found that the expression of Ngn2 protein decreased in hippocampal neurons in the acute stage after experimental stroke. Moreover, *ngn2* gene overexpression alleviated neuronal apoptosis and enhanced BDNF, p-TrkB and Bcl-2 expression, which inhibited CytC leakage from the mitochondria to the cytoplasm. Furthermore, TAT-Ngn2, which crossed the BBB and was distributed into hippocampal neurons, enhanced cognitive functional recovery; alleviated hippocampal neuronal damage and apoptosis, regulated BDNF levels and p-TrkB levels and reduced caspase-dependent apoptotic pathways. These results suggest that the downregulation of Ngn2 expression may have an important role in triggering brain injury after ischemic stroke and that TAT-Ngn2 may be an attractive therapeutic strategy in the acute stage after cerebral ischemic injury.

After years of research and extensive pathophysiological investigation of stroke, much is known in animal models about the pathways that may contribute to brain injury (Hansen-Schwartz et al., 2007; Macdonald et al., 2007). Global cerebral ischemia occurs when the blood supply to the entire or a large part of the brain is impeded (Harukuni and Bhardwaj, 2006), which may also arise from a number of clinical conditions such as cardiac arrest, drowning or systemic hypotension during surgery. This transient insult attacks vulnerable brain regions, such as the CA1 region in the hippocampus, and leads to neuronal damage (Li et al., 2017). However, the underlying mechanisms and effective therapeutic targets need to be further explored and proven.

In our previous study, we demonstrated that exogenous Ngn2 attenuated neuronal damage and improved neurological functional outcomes after focal cerebral ischemia (Deng et al., 2013). However, the underlying associated molecular mechanisms were not understood. In the present study, we found that Ngn2 expression decreased in the hippocampus and in primary cultured hippocampal neurons in the acute stage after ischemic injury, assessed both *in vivo* and *in vitro* in an experimental stroke model, which indicated that Ngn2 represents a potential valuable target molecule for stroke therapy. It has been reported that Ngn2 is a key regulator of neurogenesis in the developing telencephalon. Moreover, transplanted Ngn2-transduced NPCs showed increased cell survival in the adult rat brain and improved motor function and sensory responses against spinal cord injury (Hofstetter et al., 2005; Yi et al., 2008). Recent studies also showed that Ngn2-transduced human NPC transplantation increased cell survival and enhanced neuroplasticity, resulting in increased structural and functional recovery in a mouse model of neonatal HI brain injury (Lee et al., 2017). Due to the function of Ngn2 in CNS development, maturation, injury and repair, Ngn2 may play an important novel role in cerebral ischemic injury.

However, the injection of Ngn2 to targeted sites by gene transfection can damage brain tissue and is far from clinical application. In this study, we utilized the TAT protein, which is a promising tool for delivering a variety of full-length peptides through the plasma membrane into cells and, most importantly, across the BBB into brain tissue (Becker-Hapak et al., 2001; Doeppner et al., 2010; Dietz, 2011; Zhang et al., 2012). We found that TAT-Ngn2 not only incorporated into hippocampal neurons when added exogenously in culture medium but also crossed the BBB to deliver TAT-Ngn2 into the hippocampus after i.p. administration. In our present study, the brains were fixed by perfusion and the hippocampal neurons were fixed before staining. It has been shown earlier that PFA-fixation of tissue may lead to an artifactual relocation of proteins (Dietz and Bähr, 2005). To clearly demonstrate transduction of cells, not fixed cells have been used for immunohistochemical analysis, or cells have pretreated with protease to remove all recombinant protein attached to the cell’s exterior before lysis for Western analysis (Dietz et al., 2002). However, as we show functional effects of TAT-Ngn2 transduction, we believe that the recombinant protein reached its sites of action both after systemic application and when applied on primary neurons.

Previous studies have demonstrated protection against ischemic insult *in vitro* and *in vivo* by delivery of TAT-linked proteins. For instance, intravitreal application of Tat-Bcl-xL reduced infarct size after 90 min transient middle cerebral artery occlusion in mice by about 75%, and improved neurological deficit scores from about 1.4 to about 0.7, and reduced the number of TUNEL positive cells in the striatum after 30 min transient ischemia by over 40% (Kilic et al., 2002). Intravenous injection of Tat-Hsp70 led to an about 75% reduction in infarct volume after 45 min middle cerebral artery occlusion compared to saline treatment, concomitant with an over 60% reduction in the number of TUNEL-positive cells in the basal ganglia (Doeppner et al., 2009a,b). We achieved an 30% reduction in TUNEL-positive cells in the hippocampus by TAT-Ngn2 application after ischemia (Figure 4) and a rescue of 50% neurons after ischemia (Figure 3C), which is in the same order of magnitude of the previously published research. However, we believe that our approach is an advancement compared to those earlier studies. Bcl-xL is an anti-apoptotic protein, and heat shock proteins, including Hsp70, have been demonstrated to be overexpressed in various cancers (Dietz, 2010). One of the important advantages of TAT-mediated delivery is also one of its pitfalls: after systemic application, the cargo TAT carries is quickly delivered to all organs and tissues, and possible side effects must be particularly carefully taken into account before clinical application can be considered (Dietz and Bähr, 2004). Ngn2 that we used as a cargo is a transcription factor known to drive glial cells into neuronal differentiation. In our previous study, we found that exogenous Ngn2 attenuated neuronal damage and improved neurological functional outcomes after focal cerebral ischemia (Deng et al., 2013). In the adult organism, delivery of exogenous Ngn2 will thus likely only affect the brain. Moreover, as the application time after stroke could be reduced to only several hours, unwanted side effects may be manageable. Another advantage of our studies might be the application route via i.p., rather than intravenous injection. Our results were also consistent with our previous studies which have demonstrated that TAT fusion proteins are safe and efficient tools for treating experimentally induced brain injury (Wang Q. et al., 2008; Wang et al., 2009; Gou et al., 2011; Mi et al., 2017; Deng et al., 2018). These results demonstrated that TAT-Ngn2 can be effectively transported and incorporated into hippocampal neurons after i.p. administration.

The global cerebral ischemia correlates with neurocognitive and behavioral deficiencies (Harukuni and Bhardwaj, 2006). In particular, learning and memory impairments are the most common neurological dysfunctions observed after global cerebral ischemic injury (Sabri et al., 2013). In this study, we comprehensively evaluated recovery of learning and memory functions in the global cerebral ischemic model, in which it was found that cognitive function was deficient due to neuronal damage, degeneration and apoptosis in the acute stage after reperfusion. Evidence suggested that C57BL/6 mice are particularly susceptible to neuronal damage in the CA1 region of the hippocampus after transient global ischemia (Yang et al., 1997). The present results also showed that TAT-Ngn2 treatment improved the recovery of cognitive function and alleviated neuronal damage, degeneration and apoptosis *in vivo* and *in vitro*. This new function of Ngn2 was supported by our previous study, in which we found that exogenous Ngn2 can reduce the infarct size and improve motor function after focal cerebral ischemia by attenuating neuronal degeneration and apoptosis in the penumbra of the cortex of mice (Deng et al., 2013). These data provide evidence that TAT-Ngn2 exerted neuroprotection against ischemic cerebral injury. In Lee’s study, they found Ngn2-NPCs significantly improved neurological behaviors, decreased cellular apoptosis, and increased the neurite outgrowth and axonal sprouting in HI brain injury. In contrast, the NPCs without Ngn2 moderately enhanced axonal extension with limited behavioral recovery. Thus, they postulated that Ngn2-expressing human NPCs facilitate functional recovery after neonatal HI brain injury via their ability to secrete multiple factors that enhance neuronal survival and neuroplasticity (Lee et al., 2017). This study suggests that Ngn2 may have additional roles in the neuronal survival. So we hypothesize that Ngn2 is capable of enhancing neuronal survival in the hippocampus after global cerebral ischemia and give the notion that TAT-Ngn2 can be used as a useful approach for stroke treatment. However, in this study, we did not focus on the function of Ngn2 on neuronal differentiation and we did not test the expression of Ngn2 in neuronal progenitor cell in the hippocampus after ischemic injury, which will be detected in our future study.

Previous studies have demonstrated that the expression of BDNF, GDNF and other neurotrophic factors in the cortex or hippocampus are influenced by cerebral ischemic injury. As a traditional neurotrophin, BDNF is widely distributed throughout the CNS, which has a major role in protecting brain tissue from injury and is involved in neuronal survival, learning and memory, neuroplasticity and neurogenesis (Han et al., 2011; Jiang et al., 2016). Previous studies have also reported that TrkB is a high-affinity endogenous receptor of BDNF. The intracellular domain of the tyrosine residue is auto-phosphorylated after BDNF binding to TrkB, which subsequently activates several intracellular signaling pathways with various functions, such as alleviating cell apoptosis (Yang et al., 2018). In this study, we found that the expression of NGF or BDNF was decreased at 48 h after reperfusion. However, TAT-Ngn2 had no effect on the expression of NGF. Then, the results showed that TAT-Ngn2 enhanced BDNF and p-TrkB expression in the hippocampus at 48 h after reperfusion. Moreover, we found that TAT-Ngn2 and *ngn2* overexpression enhanced BDNF and p-TrkB expression in cultured hippocampal neurons at 48 h after OGD injury *in vitro*. However, TAT-Ngn2 had no effect on the expression of total TrkB expression. Based on the above findings, we hypothesized that BDNF/p-TrkB signaling pathways can be regulated by Ngn2 after ischemic reperfusion injury. This hypothesis has been verified by several studies. Lee et al. (2017) reported that Ngn2 protein-transduced human NPCs significantly upregulated the gene expression levels of BDNF, increased cell survival and enhanced structural and functional recovery in a mouse model of neonatal HI brain injury. In our previous study, we found that inhibition of TrkB phosphorylation increased neuronal damage and apoptosis after OGD injury (Zhao et al., 2017). These studies indicated that Ngn2 is involved in the regulation of BDNF and p-TrkB expression, which may play an important role in neuronal survival after cerebral ischemic injury. Studies have suggested that the CytC-caspase model might be responsible for cell apoptosis after neuronal damage. CytC is a soluble protein that is loosely bound to the outer face of the inner mitochondrial membrane (Cai and Jones, 1998). After ischemic injury, the mitochondrial permeability transition pore opens due to the depolarization of mitochondrial membrane potential in apoptotic cells. Then, CytC is released from the mitochondria into the cytoplasm (Liu et al., 2017). The cytoplasm CytC activates effector caspase-3 to induce cell apoptosis. In addition, cleaved caspase-3 is considered an executioner molecule in the pathogenesis of apoptosis. To ascertain that the isolated fractions were indeed enriched for the mitochondrial or cytoplasmic compartments, respectively, markers were used as described (Guo et al., 2017; Kong et al., 2018; Li et al., 2018). By Western blotting, the mitochondrial marker COX IV was only detected in the mitochondrial fraction, but not in the cytoplasmic fraction (data not shown). The cytoplasmic marker β-actin was only detected in the cytoplasmic fraction, but not in the mitochondrial fraction (data not shown), demonstrating that our fractionation method highly enriched mitochondrial and cytoplasmic proteins. In our research, the results of western blot analysis showed low levels of CytC in the cytoplasm and high protein levels of CytC in the mitochondria after TAT-Ngn2 or *ngn2* overexpression treatment in both *in vivo* and *in vitro* ischemic models. Moreover, levels of the pro-apoptotic cleaved caspase-3 decreased after TAT-Ngn2 or *ngn2* overexpression treatment after reperfusion injury. These observations were consistent with previous studies (Deng et al., 2014; Liu et al., 2017; Zhao et al., 2017). In addition, Bcl-2 downregulation and Bax up-regulation also contribute to CytC release from the mitochondria and result in the activation effect of caspase-9 on caspase-3, finally promoting apoptosis (Guégan et al., 2006; Song et al., 2010; Ji et al., 2012). In fact, it has been reported that Bcl-2 has the function in preventing apoptosis, while Bax serves as a pro-apoptotic molecule (Mao et al., 2015). Another study also showed that huperzine A can attenuate apoptosis in hippocampal HT22 cells by activating BDNF/TrkB to enhance Bcl-2 expression and inhibit Bax expression (Mao et al., 2016). Furthermore, inhibiting the phosphorylation of TrkB decreased Bcl-2 expression and increased Bax expression, which induced mitochondria to release CytC and activate caspase-3, ultimately leading to increased cell apoptosis activity in the OGD model, as demonstrated in our previous study (Zhao et al., 2017). In the present study, the pro-apoptotic cleaved caspase-3 and Bax decreased and the anti-apoptotic protein Bcl-2 increased after treatment with TAT-Ngn2 or *ngn2* overexpression after reperfusion injury, which was consistent with previous studies. These results confirmed that Bax and Bcl-2 played an important role in CytC release and caspase-3 activation after brain reperfusion injury in accordance with previous studies. These findings also suggested that the neuroprotection of TAT-Ngn2 against stroke might have involved the modulation of BDNF-TrkB signaling that regulates caspase-dependent and mitochondrial neuronal apoptotic pathways.

It is not clear what molecules regulate the expression levels of Ngn2 in hippocampal neurons after ischemic reperfusion injury. During neurogenesis, Wnt signaling pathways positively regulate Ngn2 expression, whereas Notch signaling pathways negatively regulate Ngn2 expression levels (Yoon and Gaiano, 2005; Machon et al., 2007; Shimojo et al., 2008). However, whether Notch or Wnt signaling pathways are involved in the regulation of Ngn2 expression in the cortex or hippocampus of mature neurons after ischemic reperfusion injury needs further study. It is also unknown how Ngn2 regulates BDNF expression. If future work reveals that BDNF is expressed in the Ngn2-dependent manner in mature neurons after ischemic reperfusion injury, new pathways for Ngn2 and BDNF rescue of neuronal apoptosis may emerge. On the other hand, most of the Western blot bands in our study were overexposed, so that the generated signals are not at all proportionally strong to the amount of protein present, which was at best qualitative, not quantitative.

In conclusion, in accord with our results, the present study suggested that downregulation of Ngn2 in the CA1 region of the hippocampus decreased BDNF expression and TrkB phosphorylation, which inhibited Bcl-2 expression and enhanced CytC leakage from the mitochondria to the cytoplasm, aggravating neuronal apoptosis after global cerebral ischemic injury. Furthermore, TAT-Ngn2 exerted protective effects contributing to cognitive functional recovery and to alleviate hippocampal neuronal damage and apoptosis via activating BDNF and p-TrkB regulating caspase-dependent and mitochondrial neuronal apoptotic signaling pathways. These results highlighted that TAT-Ngn2 may be an attractive therapeutic strategy in the acute stage after cerebral ischemic injury and may be an efficient therapeutic agent for treating CNS diseases.

## Author Contributions

BD, LH and HS designed the experiments. YZ, JW, JD, BL and JL performed the experiments. YZ, XG and JD performed the data analyses. BD, LH and HS wrote the article.

## Conflict of Interest Statement

The authors declare that the research was conducted in the absence of any commercial or financial relationships that could be construed as a potential conflict of interest.
